# An interesting presentation of a rare association of the Wilkie and Nutcracker syndromes

**DOI:** 10.1016/j.radcr.2023.05.020

**Published:** 2023-05-29

**Authors:** Barbara Brogna, Andrea La Rocca, Vera Giovanetti, Marta Ventola, Elio Bignardi, Lanfranco Aquilino Musto

**Affiliations:** a Department of Interventional and Emergency Radiology “San Giuseppe Moscati Hospital”, Contrada Amoretta, 83100, Avellino, Italy; b Division of Radiology, Università degli Studi della Campania Luigi Vanvitelli, 80138, Naples, Italy; c Department of Medicine and Health Science, University of Study of Molise, C/da Tappino, Campobasso 86100, Italy; d Radiology Unit, Cotugno Hospital, Via Quagliariello, 54, Naples 80131, Italy

**Keywords:** Aortic mesenteric syndrome, Wilkie syndrome, Nutcracker syndrome, CT, Arterial hypertension

## Abstract

Superior mesenteric artery syndrome also known as Wilkie's syndrome (WS) and Nutcracker syndrome (NCS) are 2 rare vascular syndromes characterized by the reduction of the aortomesenteric space. In the WS the reduction of the aortomesenteric angle leads to compression of the third portion of the duodenum. In the NCS the reduced aortomesenteric space usually causes a left renal vein (LVR) entrapment and the clinical presentation is a left flank pain, micro/macrohematuria and proteinuria. Arterial hypertension can be an unusual manifestation of the NCS. Herein, we describe the case of a 37-year-old woman with a history of breast cancer and abdominal subocclusion, with a recent onset of arterial hypertension whose enhanced computed tomography (CT) showed a reduced angle between the abdominal aorta and superior mesenteric artery with the CT findings of both the WS and NCS.

## Introduction

Superior mesenteric artery syndrome, also known as Wilkie's syndrome (WS), is a rare cause of acute abdomen in the emergency setting presenting with abdominal obstruction [1], [2], [3], [4], [5], [6]. This condition is characterized by a decreased angle between the superior mesenteric artery and the abdominal aorta, which further causes an extrinsic compression of the third portion of the duodenum, preventing the passage of intestinal contents and leading to an upper gastrointestinal obstruction. This results in chronic, intermittent, or acute duodenal obstruction [1,^2^,4]. An aortomesenteric angle less than 25° or an aortomesenteric distance less or equal to 8mm are the most frequently cited diagnostic criteria [1], [2], [3], [4]. The vascular angles and distances are measured on the computed tomography (CT) sagittal and axial reformation. The incidence of this condition is rare, and it was found in approximately 0.013%-0.3% of cases in the general population [5], especially among women. This syndrome can be congenital or acquired. The acquired form is the most common and it is caused by a rapid reduction of the fat surrounding the abdominal aorta and superior mesenteric artery. This condition is usually common in anorexic or oncologic patients [3]. Nutcracker syndrome (NCS) is defined as a decreased angle between the superior mesenteric artery and the aorta with extrinsic compression of the left renal vein (LRV) and the clinical presentation is a left flank pain, micro/macrohematuria and proteinuria [3,^4^,[6], [7], [8]. If asymptomatic, left renal vein entrapment is called the Nutcracker phenomenon or anatomy [8]. The term of NCS is used for patients with clinical symptoms associated with Nutcracker anatomy [3,^4^,[6], [7], [8]. The WS and NCS share the same pathogenesis and can be associated. In fact, in WS, the reduction of the aortomesenteric space can be associated with LRV compression. However, the simultaneous combination of these vascular alterations is rare.

## Case presentation

**Fig. 1:**
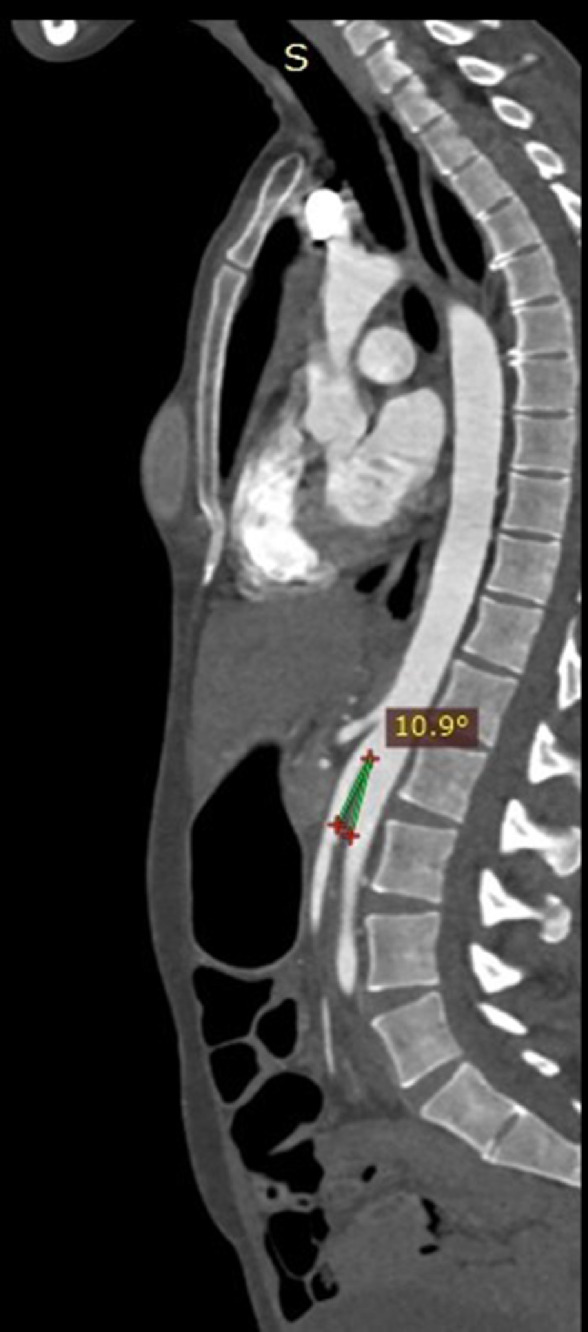
This figure shows the angiographic phase on the enhanced CT with the reduced angle between the abdominal aorta and the superior mesenteric artery.

**Fig. 2:**
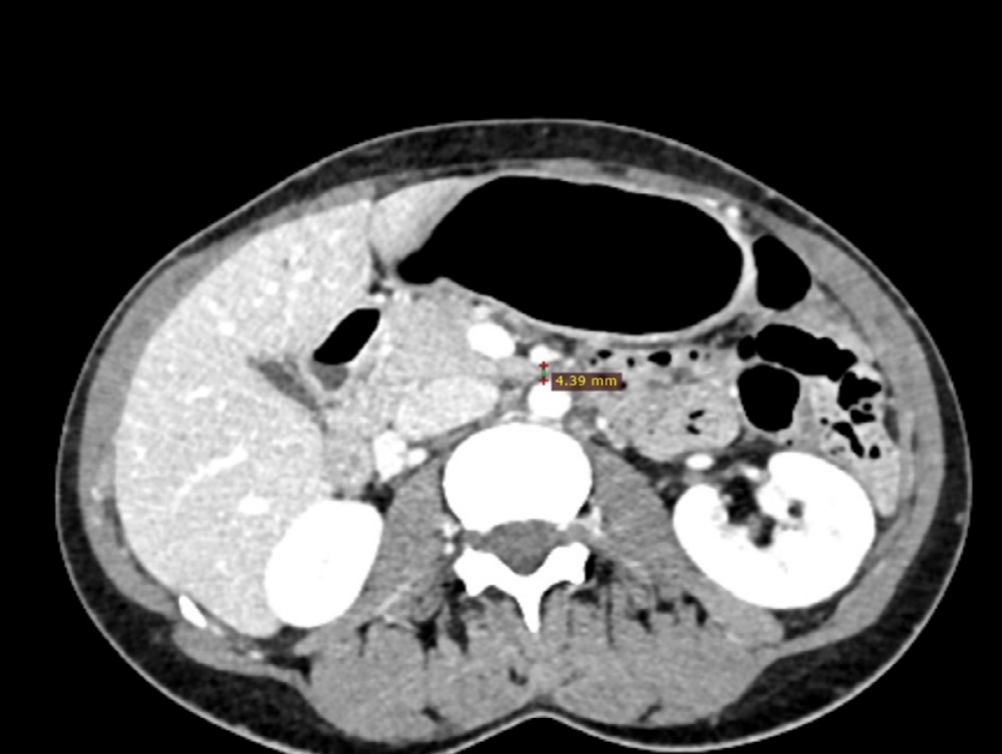
This figure shows the reduction of the aortomesenteric space (4.39 mm) with the compression of the third portion of the duodenum and a mild distension of the stomach.

A 37-year-old woman, who had undergone a bilateral mastectomy for breast cancer 2 years earlier, came to our Institution complaining of frequent abdominal pain with subocclusive episodes, nausea, and vomiting and for regular follow-up for the previous surgery. Her mother had died from colic and pancreatic cancer. She had a history of 3 cesarean deliveries, the last one in 2016. Following the last cesarean, she began to suffer from abdominal pain and rapid weight loss (BMI 16.4). In 2019, the patient was admitted to the emergency for a subocclusive episode where a reduction of the aortomesenteric angle was described. However, no other alterations were reported. The patient was treated conservatively with periodic insertion of a nasogastric tube to decompress the stomach, a high-protein diet and parenteral nutritional supplements. However, the patient reported recent episodes of hypertension (PA 140/100 mm Hg) with frequent headaches. The clinicians who hypothesized a psychiatric condition did not consider these symptoms with due attention. The patient had no family history of hypertension. Her family doctor considered prescribing laboratory examinations. The results of the urine screening test showed the presence of microhematuria with an increased value of the protein/creatinine ratio (300 mg/gr creatinine), an increased value of albumin (80 mg/L), and an increased value of albumin/creatinine ratio (150 mg/gr creatinine). The other laboratory results were in the normal range. The enhanced thoracic abdominal computed tomography (CT), confirmed the reduction of the aortomesenteric angle (about 11°) (a normal value ranging from 25° to 60°) with compression of the third portion of the duodenum and a mild distended stomach but with no sign of intestinal occlusion (Figs. 1, and 2). It was also described a reduced aortomesenteric distance (Fig. 2).

**Fig. 3:**
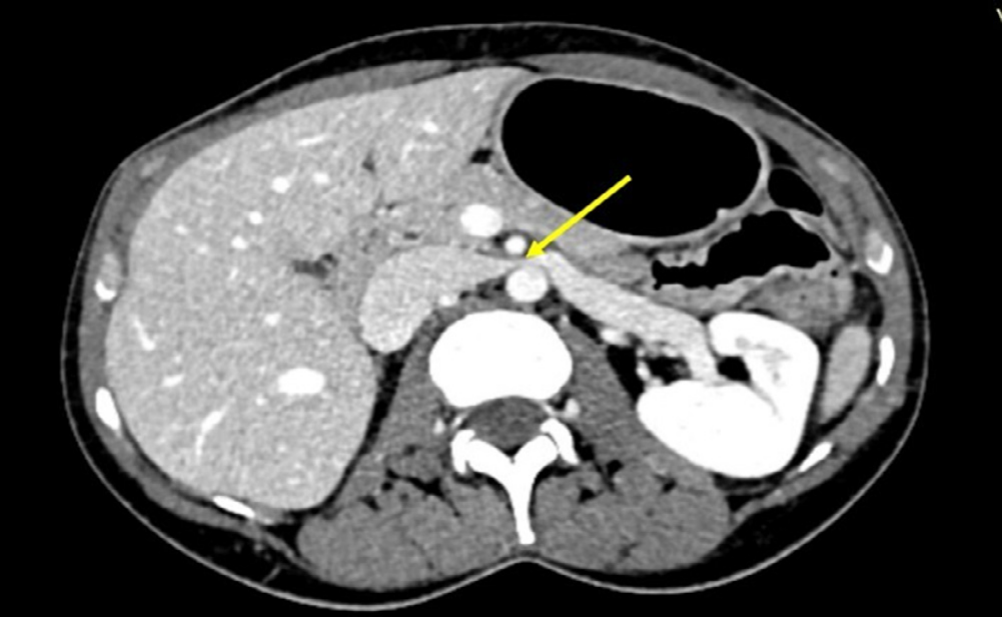
This image describes the compressed left renal vein (LVR) between the aortomesenteric space (yellow arrow) with the beak sign on the axial plane.

**Fig. 4:**
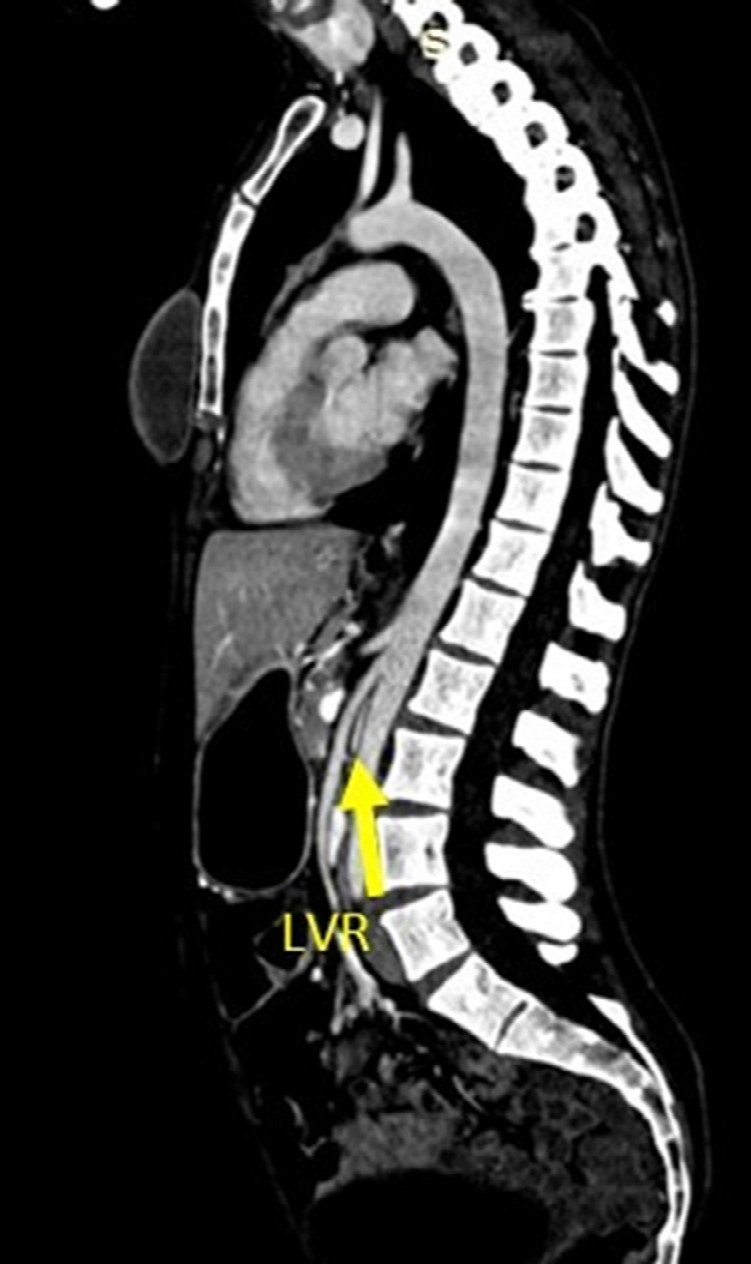
The compressed left renal vein (LVR) (yellow arrow) between the abdominal aorta and the mesenteric artery on the sagittal plane.

There was, however, evidence of entrapment of the left renal vein (LVR) (Figs. 3, and 4) with evidence of the beak sign on CT (Fig. 3).

Following the imaging results, a diagnosis of combined WS with NCS was made. Subsequently, the patient was referred to a nephrologist in consideration of a possible association of the recent onset of her arterial hypertension with the Nutcracker syndrome which resulted from the imaging, and also for correct management. A follow-up with a renal Doppler ultrasound was also planned.

## Discussion

The WS and the NCS are 2 rare vascular syndromes characterized by the reduction of the aortic-mesenteric space [3,^4^,[6], [7], [8]. Although the WS and NCS share the same pathogenesis, the combination of these 2 vascular alterations is rare. In the WS there is a decreasing angle between the superior mesenteric artery and the abdominal aorta (<22°) (normally value range between 38° and 65°) with an aortomesenteric distance reduction (<8 mm) (normal aortomesenteric distance is 10-28mm) [2,3] that causes compression on the third portion of the duodenum leading to chronic, intermittent, or acute, complete or partial, duodenal obstruction. However, the most typical clinical manifestations may be nonspecific and may include postprandial epigastric pain, nausea, vomiting, and early satiety.

Radiologists play a pivotal role in the diagnosis of vascular syndromes because they can address a correct diagnosis. In fact, the WS, although is rare, can be a cause of abdominal occlusion in young women. Doppler ultrasound (US) is considered the first-level examination for the evaluation of vascular compression syndromes [2,^9^,10]. However, patients with WS usually come to the emergency with an upper abdominal occlusion and the diagnosis is mostly made on CT [1,^3^,11]. Through multiplanar reconstruction (MPR) on CT, radiologists can easily make the correct measurements and address the correct diagnosis. Ancillary signs on CT are a distended stomach with compression of the third duodenal portion [2,3]. In the NCS the reduction of aortomesenteric space causes a LVR entrapment. However, the syndrome can be divided into 3 types based on the anatomy: anterior, posterior, and mixed [8,12]. Anterior NCS is characterized by compression of LRV due to a decrease of the aortomesenteric space [8,12]. Posterior NCS results from retro aortic compression of LRV between the abdominal aorta and the vertebral column [8,12]. The mixed form is the most uncommon one and is characterized by both alterations [13]. NCS can manifest with typical and atypical symptoms. Typical symptoms include gross hematuria and orthostatic proteinuria, with or without flank pain. Atypical symptoms include fatigue, flank/abdominal pain, orthostatic intolerance, dysmenorrhea and dyspareunia in women, and varicocele in men [8,12]. Although hypertension is not generally included in the traditional clinical manifestations of NCS, it has been reported in rare cases as the first manifestation of NCS [14,15]. The first line imaging tool to follow or diagnose the NCS is usually the Doppler US which allows us to obtain an estimate of LRV stenosis degree [3,^9^,12]. On CT the entrapment of the renal vein between the aortomesenteric space is visualized as the “beak sign” [2]. The treatment of these rare vascular syndromes when asymptomatic or mild symptomatic is usually conservative and consists of a high-calorie protein diet with the aim to restore a normal aortomesenteric space [1,^4^,12]. Other therapeutic approaches are surgical or endovascular treatments and are reserved for symptomatic patients [1,13]. In our case, the patient was not initially followed for a possible association of the WS and NCS and clinicians undervalued her symptoms. Therefore, Radiologists have an important role in the diagnosis and should pay attention to the possible combination of the NCS and WS. On the other side, clinicians should not overlook this combination, despite their rarity.

## Conclusion

Since WS and NCS are rare vascular conditions, clinicians may not consider the symptoms with due attention. However, these syndromes can be the root cause of abdominal pain in young women and this phenomenon should not be overlooked. Radiologists should pay attention to the symptoms and consider the possible association of the WS to the NCS. A first clinical presentation of the NCS in young patients may consist of arterial hypertension with headache, thus these conditions should always be further investigated.

## Author's contribution

All listed authors must have made a significant scientific contribution to the research in the manuscript

## Patient consent

Written informed consent of the patient has been obtained to publish this case report and it will be available on Journal Request
